# QTL mapping for different resistant starch subtypes identified a superior haplotype balancing high RS content and relatively good eating and cooking qualities in rice

**DOI:** 10.3389/fpls.2026.1763165

**Published:** 2026-01-27

**Authors:** Cheng Liang, Yuesi Bu, Haoyang Xu, Xuemei Ma, Xueying Zhang, Tian Hu, Xunchao Xiang, Yungao Hu, Liang Xu

**Affiliations:** 1 College of Life Sciences and Agri-forestry, Southwest University of Science and Technology, Mianyang, China; 2 Rice Research Institute, Southwest University of Science and Technology, Mianyang, Sichuan, China; 3 Institute of Crop Sciences, Chinese Academy of Agricultural Sciences, State Key Laboratory of Crop Gene Resources and Breeding, Beijing, China

**Keywords:** eating and cooking qualities, QTL mapping, resistant starch, rice (*Oryza sativa* L.), *Wx^a^-SSIIa^G-TT^* haplotype

## Abstract

Resistant starch (RS) plays an important physiological role in maintaining human health. However, increasing RS content in rice often comes at the cost of deteriorating its eating and cooking qualities (ECQs). In order to address this conflict, we conducted co-localization quantitative trait locus (QTL) analysis for RS in raw rice flour (RSm), cooked rice (RSc), retrograded rice (RSr) along with correlation analysis between RS and ECQs, using recombinant inbred line (RIL) populations derived from a cross of CG133R and Javanica 22. A total of 33 QTLs associated with RSm, RSc, RSr, RSa, and RSb were identified. These included two major QTLs on choromosome 6 (*Wx* and *SSIIa*), and several novel minor-effect QTLs such as *q2ERSc3.2*, *q2ERSb5.1*, and *q2ERSb9.1* on choromosome 3, 5 and 9, respectively. *Wx* accounted for 27.34%, 64.16%, 68.07%, 29.95%, and 39.62% of the phenotypic variance for RSm, RSc, RSr, RSa (RSm-RSc), and RSb (RSr-RSc), respectively. Meanwhile, *SSIIa* explained 42.42%, 17.82%, 14.09%, and 51.16% of the phenotypic variance for RSm, RSc, RSr, and RSa. Furthermore, the thermal and retrogradation properties demonstrated positive correlations with RSm, but negative correlations with RSc and RSr, which was attributed to the differential regulation of *Wx* and *SSIIa*. *Wx^a^-SSIIa^G-GC^* regulated high RSm and RSa, while *Wx^a^-SSIIa^G-TT^* significantly increased RSc and RSr. Notably, *Wx^a^-SSIIa^G-TT^* haplotype improved the rice ECQs by reducing gelatinization temperature, preventing retrogradation and enhancing viscosity properties. Thus, this study identified an excellent haplotype, *Wx^a^-SSIIa^G-TT^*, which enhanced RSc and RSr and improved rice ECQs, providing useful information for breeding high-RSc rice with a relative superior quality.

## Introduction

Rice (*Oryza sativa* L.) is an important staple food that sustains more than half of the world’s population and provides essential caloric and nutrition (Tiozon et al., 2023). Driven by evolving lifestyles and higher living standards, the type 2 diabetic population is rapidly expanding. A high resistant starch (RS) diet has been shown to help prevent diabetes while reducing calorie intake, potentially aiding in weight management efforts (Luo et al., 2023). Based on distinct physicochemical properties and botanical origins, RS is divided into five subtypes (RS1 - 5) (Xia et al., 2018). Natural rice primarily contains RS2, while RS5 becomes predominant after cooking, and RS3 is generated in gelatinized starch through cooling and retrogradation (You et al., 2022). RS2 is densely organized in a radial configuration within native starch granules, effectively limiting accessibility to digestive enzymes (particularly amylases). It is therefore primarily found as granular resistant starch in high-amylose cereals (Shen et al., 2022). RS3 is a type of retrograded starch characterized by a semi-crystalline, double-helical structure formed through the reorganization of amylose and amylopectin during the chilling of cooked rice (Alhambra et al., 2019). RS5, formed through the complexation of lipids with amylose within starch granules, inhibits granule expansion and confers resistance to enzymatic hydrolysis (Zhou et al., 2016). Therefore, the differences in RS content among raw milled rice (RSm), hot cooked rice (RSc), and retrograded rice (RSr) are associated with the distinct regulatory genes and complex genetic control mechanisms involved in their biosynthesis (You et al., 2022).

The biosynthesis of RS is regulated by coordinated interactions among starch synthesis enzymes. Genetic variations in the genes encoding granule-bound starch synthase (GBSS), soluble starch synthase (SS), and starch-branching enzyme (SBE) have been shown to significantly impact RS accumulation in major cereal crops including rice, maize (*Zea mays*), and wheat (*Triticum aestivum*) (Zhou et al., 2016; Shen et al., 2022). Numerous studies have demonstrated that part of *starch-synthesis-related genes* (*SSRGs*) played crucial roles in regulating RS content in rice. Four quantitative trait loci (QTLs) associated with RS content were identified by a genome-wide association study (GWAS) in rice grains, with each explaining 10-13% of the phenotypic variance. Two QTLs were mapped to chromosome 6, corresponding to the *Waxy* (*Wx*) and *soluble starch synthase IIa* (*SSIIa*) loci, respectively. The other two were localized on chromosomes 8 and 9, in proximity to the genes encoding SBEI and the small subunit I of ADPase, respectively (Bao et al., 2017). Meanwhile, researcher revealed that via precisely regulating the *cis*-regulatory element of *SBEIIb*, the content of RS in the rice endosperm could be significantly increased (Yang et al., 2025). In rice endosperm, the *Wx* gene encodes GBSSI, which is involved in the synthesis of amylose. Multiple *Wx* alleles (including *Wx^a^*, *Wx^b^*, *Wx^lv^*, *Wx^op/hp^*, *Wx^mq^*, *Wx^la/mv^*, and *Wx^mp^*) have been identified to regulate the synthesis of varying amylose content (AC) (Zhang et al., 2021; Zhou et al., 2021). Rice varieties carrying the *Wx^a^* allele associated with high AC have a higher RS content compared to those carrying the *wx* allele (loss of function and no amylose synthesis) (You et al., 2022). In rice, *SSIIa* significantly influences RS formation. Specifically, the *indica* allele *SSIIa^G-GC^* promotes higher RS content than the *japonica SSIIa^G-TT^* allele. This difference arises since the highly active SSIIa enzyme enhances starch crystallinity, which in turn increases enzymatic resistance and raises RS levels (Bao et al., 2017; You et al., 2022). Moreover, extensive research on mutant materials has demonstrated that allelic variations in specific *SSRGs* resulted in higher RS content. The *b10* mutant, derived from *indica* rice R7954, contains a G-to-A mutation at the 3´splice site of the fifth intron in *SSIIIa*, which led significantly increases on RS content (Zhou et al., 2016). The genetic study demonstrated that the high RS phenotype in the *rs4* mutant was driven by the synergistic interaction of loss-of-function mutations in both *SSIIIb* and *SSIIIa*, combined with the presence of the *Wx^a^* allele (Wang et al., 2023). The *sbe3-rs* mutant, characterized by a single amino acid substitution in the SBEIIb enzyme, is associated with elevated RS content in Jiangtangdao 1 (Yang et al., 2016). In addition, our previous study utilized targeted genome association analysis to reveal significant genetic effects of *Wx*, *SSI*, *SSIIa*, *SSIIb*, *Pullulanase* (*PUL*), and *SSIVb* on diverse RS types in rice (Liang et al., 2024). However, current research on RS regulatory genes in rice mainly focuses on SSRGs, while the study of new regulatory sites discovered through QTL mapping is relatively limited. Furthermore, it is elusive to identify favorable haplotypes for different types of RS in rice and to investigate the co-regulatory patterns among these genes.

The formation of RS is governed by both the intrinsic properties of starch including granule architecture, crystalline structure, and amylose-to-amylopectin ratio, and the presence of non-starch constituents such as proteins, lipids, and sugars (Tian and Sun, 2020; Shen et al., 2022). Amylose is indispensable for RS formation, as evidenced by a highly significant positive correlation between RS content and AC (Kong et al., 2015). Besides, previous studies have shown that RS content significantly correlated with eating and cooking qualities (ECQs). The consistency viscosity (CSV), setback viscosity (SBV), and pasting temperature (PaT) of viscosity parameters (PVPs) showed notable associations with RS content (Chen et al., 2017). In addition, RS2 exhibited a positive correlation with both hot paste viscosity (HPV) and cool paste viscosity (CPV), whereas PVPs showed a strong negative correlation with RS3 (Ding et al., 2019). However, high AC leads to a deterioration in the ECQs of rice. Therefore, further analyzing the relationship between RS content and other indicators of rice ECQs such as gel consistency (GC), gelatinization temperature (GT), and PVPs is of great significance for improving the ECQs of rice varieties with high RS. Concurrently, combining the identification of favorable haplotypes in major genes controlling RS and ECQs with molecular breeding, making it achieve multi-gene pyramiding to increase RS and simultaneously enhance rice quality. Additionally, identifying novel genetic loci that regulate RS through QTL mapping, combined with molecular design breeding to mitigate the genetic influence of *Wx* on RS, represents a pivotal strategy for enhancing quality in high-RS rice varieties. This study employed recombinant inbred lines (RILs F_8_ and F_11_) to conduct a systematic genetic analysis of multiple RS subtypes, and integrated correlation with multidimensional ECQs, aiming to systematically identify genetic targets that overcome the “high RS vs. low quality” trade-off.

## Materials and methods

### Materials planting and flours preparing

A total of 103 rice lines were derived from the RILs constructed by the hybridization of CG133R (*Oryza sativa* ssp. *indica*, a restorer line, AAC ≈ 27%) and Javanica22 (*Oryza sativa* var. *Glutinosa*, a natural variation derived from Xiangdali, AAC ≈ 0.7%). The two populations of F_8_ and F_11_ obtained from planting in 2018 and 2021 respectively were taken as the research materials. All rice lines were cultivated under natural summer conditions at the experimental field of Southwest University of Science and Technology’s agricultural base (Sichuan, China), following standard agronomic practices. At maturity, three individual plants per line were harvested as biological replicates for further analysis. After drying at a constant temperature of 42°C for 96 hours, the seeds were stored at room temperature for 3 months to equilibrate moisture content. Then, using a dehuller (TR-200 Kett, Tokyo, Japan) to mechanically dehull the seeds. The resulting brown rice was polished with a polisher (Pearlest, Kett, Tokyo, Japan), followed by milling into flour through a 100-mesh sieve (0.15mm) in a Laboratory Mill (LM3100, Petern, Malmö, Sweden).

### Genomic DNA extraction and genotyping

The genomic DNAs of 103 rice lines were extracted using the cetyltrimethylammonium bromide technique from fresh leaves (Xu et al., 2020). To conduct genetic analysis and screen for genetic differences among parents, 833 pairs of molecular markers evenly distributed across 12 chromosomes were selected from SSR markers developed on the Gramene website (https://www.gramene.org/) and highly polymorphic SSR markers, CAPS markers, and In-Del markers reported in previous studies (Singh et al., 2010; Tian et al., 2010; Yan et al., 2010). The physical locations of molecular markers on chromosomes are marked in **Supplementary Figure S1**. Subsequently, a total of 123 molecular markers that exhibited polymorphisms between the parents were utilized to genotype the F_8_ and F_11_ populations and to perform QTL mapping. Genotyping was performed using agarose gel electrophoresis method and the polymerase chain reactions (PCR) ran on an Eppendorf Thermal Cycler (Mastercycler^®^ nexus GSX1, Germany). The PCR systems and procedures used for different molecular markers were consistent with the method adopted by (You et al., 2022). Amplified products of PCR were detected on a 3% agarose gel in 0.5 × Tris-Borate EDTA (TBE) buffer using GreenView (Applied BioProbes, Rockville, MD, USA).

### Determination of the content of different types of RS

Three different RS (RSm, RSc, and RSr) were measured with the same sample. The preparation of cooked rice followed the protocol outlined by Zhou et al. (2016). Specifically, rice flour and water were mixed in a centrifuge tube at a ratio of 1:1.8, boiled in water for 15 minutes, and then left in hot water for an additional 10 minutes before being removed and allowed to cool to room temperature. For retrograded rice, the cooking method was the same as that for cooked rice. After cooking and cooling to room temperature, the retrograded rice was stored at 4 °C for 7 days prior to measurement. The determination of resistant starch content was conducted according to the method described by McCleary et al. (2002). Each starch sample was measured in duplicate. Furthermore, the differences between RSm and RSc, RSr and RSc were calculated, RSa = RSm - RSc, RSb = RSr - RSc. RSa and RSb respectively represent RS2 and RS3 in rice (You et al., 2022).

### Determination of ECQs

The apparent amylose content (AAC) was determined using the spectrophotometric method at a colorimetric wavelength of 620 nm, in accordance with the Chinese Ministry of Agriculture’s standard NY/T 2639-2014, and each sample was measured twice. GC was measured in accordance with Chinese national standards NY/T147–88 in duplicate, and a third measurement was made if the difference of the two measurements exceeded 7 mm. The thermal properties of starch were determined using the differential scanning calorimetry (DSC) produced by METTLER TOLEDO. Weigh 5.0 mg of the sample and transfer it into a specialized crucible. Add double the volume of ddH_2_O (double-distilled water), then seal the crucible. After refrigeration it at 4°C for more than 12 hours, equilibrate the sample at room temperature for 1 h prior to instrumental analysis. The DSC thermal program was configured with a heating ramp from 30°C to 100°C at 10°C/min. By analyzing the DSC curves, the onset temperature (To), peak temperature (Tp=GT), conclusion temperature (Tc) and the enthalpy change (ΔH) of the sample can be obtained. The gelatinized starch was stored at 4°C for 7 days to induce retrogradation. The retrograded starch was then analyzed using the same DSC thermal program to determine its retrogradation properties, including the retrograde onset temperature (Tor), retrograde peak temperature (Tpr), retrograde conclusion temperature (Tcr), and retrograde enthalpy change (ΔHr). The retrogradation rate (R%) was calculated as R% = ΔHr/ΔH × 100%, where ΔH represents the initial gelatinization enthalpy. All thermal parameters were measured in duplicate for each sample.

### Determination of viscosity properties

The viscosity properties of rice flour were characterized by analyzing the viscosity profile using a Rapid Visco Analyzer (RVA4500, Newport Scientific, Warriewood, Australia). A mixture of 3.0 g rice flour (14% moisture basis) and 25.0 g deionized water was prepared in an aluminum RVA canister. The viscosity measurement was performed following both the manufacturer’s protocol and the American Association of Cereal Chemists’ standard method: AACC61 - 02. The RVA profile provided measurements of peak viscosity (PKV), hot paste viscosity (HPV), CPV, PaT, and peak time (PeT). Three derived parameters were subsequently calculated: breakdown viscosity (BDV = PKV - HPV), SBV = CPV - PKV, and CSV = CPV - HPV. All measurements were performed in duplicate for each sample.

### Statistical analyses

one-way analysis of variance (ANOVA) was performed using IBM SPSS Statistics 26. Origin 2022 was employed for correlation analysis and graphical visualization. The genetic linkage map was constructed using the “MAP” module of QTL IciMapping 4.2, while QTL mapping and multi-environment QTL (MQTL) analyses were conducted using the “BIP” (Biparental Populations) and “MET” (Multi-Environment Trials) functions, respectively. The LOD threshold was set at 2.0, following Wang et al. (2007) and QTLs were named according to the nomenclature system proposed by McCouch and Cgsnl (2008).

## Results

### Distribution of RS content between two generations in the RIL population

**Figure 1:**
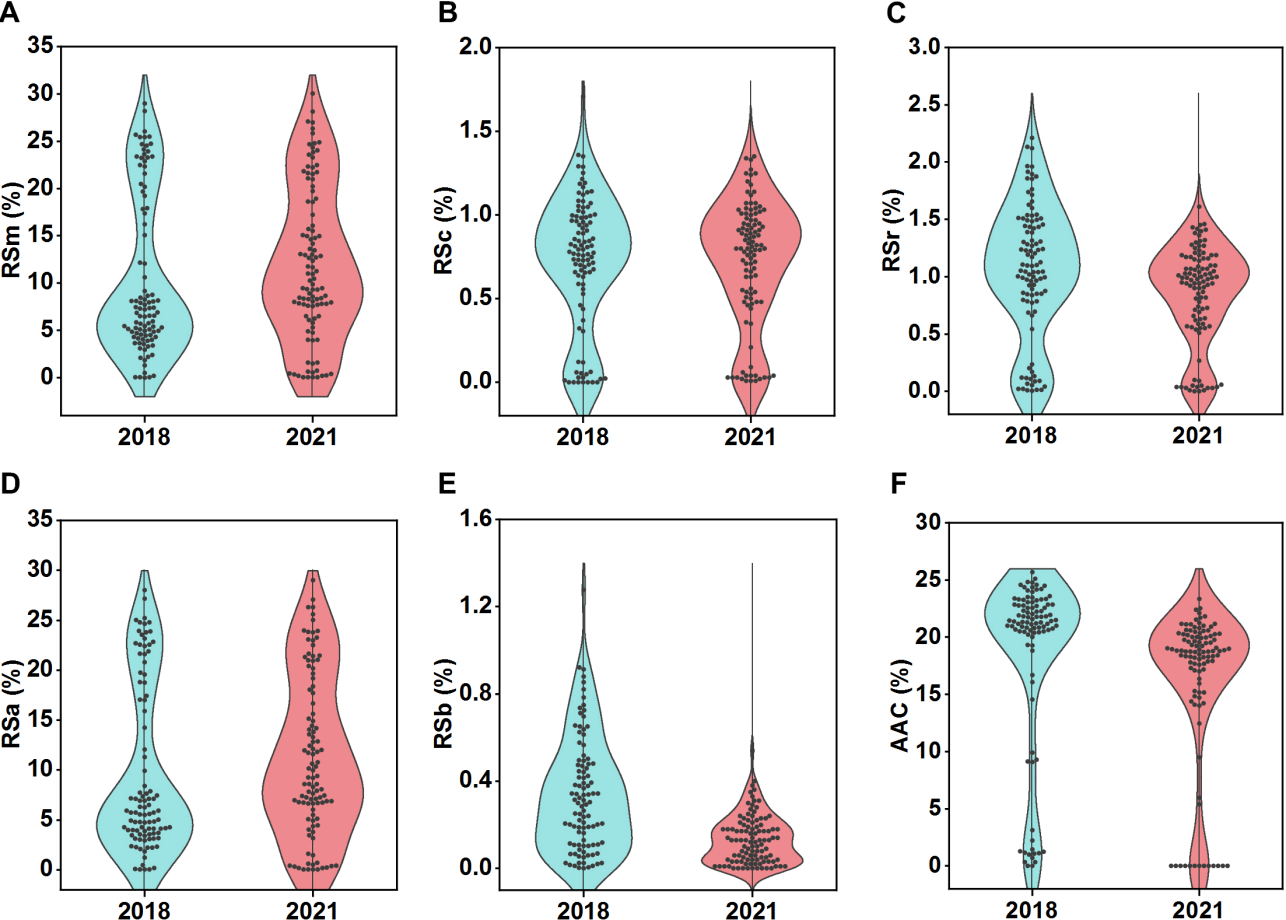
Distribution of different RS types in RILs across two generations. **(A)** RSm, resistant starch content in milled rice; **(B)** RSc, resistant starch content in cooked rice; **(C)** RSr, resistant starch content in retrograded rice; **(D)** RSa is equal to RSm–RSc; **(E)** RSb is equal to RSr–RSc. **(F)** AAC, apparent amylose content.

**Figure 2:**
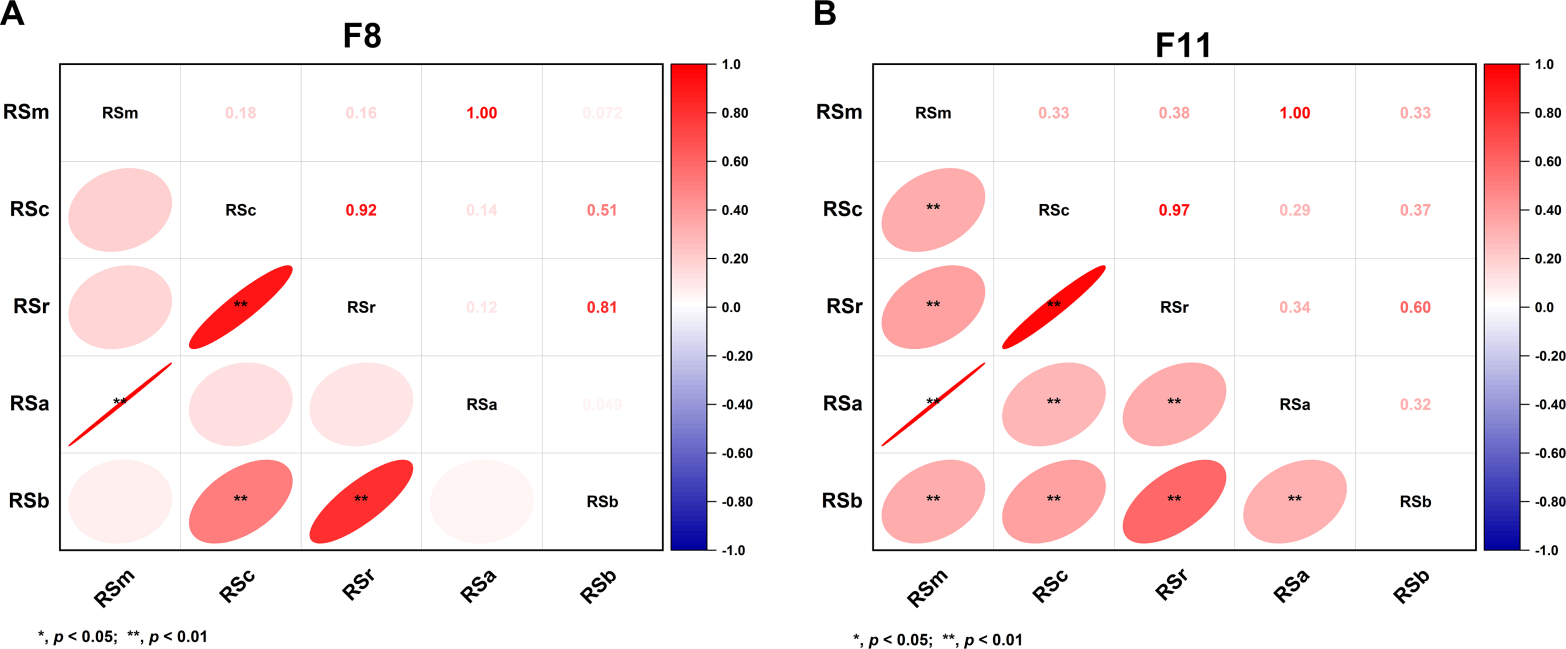
Correlation analysis of different RS types of RILs across two generations. **(A)** F_8;_**(B)** F_11_. RSm, RS content in raw milled rice; RSc, RS content in cooked rice; RSr, RS content in retrograded rice; RSa is equal to RSm–RSc; RSb is equal to RSr–RSc.

We first analyzed the RS content variations between parental lines and their RILs in 2018 (F_8_) and 2021 (F_11_). The results revealed significant diversities on all RS types (RSm, RSc, RSr, RSa, and RSb) between the parents (*P* < 0.01) in equal environment, except for RSb in 2021 (**Supplementary Figure S2**). And all five RS types exhibited wide variations within the RIL populations, with coefficients of variation ranging from 47.19% to 83.73% (**Supplementary Table S1**). This substantial phenotypic diversity, indicative of rich genetic polymorphisms, made the population well-suited for QTL mapping. Further analysis revealed that except for RSb, all other RS types maintained stable across two generations, suggesting its heightened sensitivity to environmental factors (**Figure 1**). Variance analysis revealed that all RS types were affected by genotype-environment interaction effects (*P* < 0.001). However, the RSa, RSm, RSc, and RSr exhibited broad sense heritability exceeding 0.7, indicating that these four RS types were predominantly controlled by genotype. However, RSb’s broad sense heritability was below 0.5 (**Table 1**), suggesting it was mainly regulated by environmental factors and genotype-by-environment interactions. Correlation analysis of five RS in different years indicated that the associations between RSm and RSa, RSc and RSr, and RSb with both RSc and RSr remained stable, exhibiting highly significant positive correlations in both generations (r > 0.5) (**Figure 2**).

**Table 1:** Variance analysis of different types RS contents of RILs across two generations.

Trait	Source of variation	SS	MS	*F*-value	*P*-value	Heritability
RSm	Genotype	32351.00	404.39	2302.11	0.0000	0.8927
	Environment	275.04	275.04	1565.73	0.0000	
	G×E	1915.35	23.94	136.30	0.0000	
RSc	Genotype	61.67	0.77	493.74	0.0000	0.8330
	Environment	0.00	0.00	0.78	0.3776	
	G×E	5.92	0.07	47.40	0.0000	
RSr	Genotype	103.74	1.30	580.53	0.0000	0.7623
	Environment	4.62	4.62	2068.14	0.0000	
	G×E	15.79	0.20	88.37	0.0000	
RSa	Genotype	31820.00	397.75	2222.23	0.0000	0.8933
	Environment	273.88	273.88	1530.17	0.0000	
	G×E	1870.88	23.39	130.66	0.0000	
RSb	Genotype	11.23	0.14	41.57	0.0000	0.3639
	Environment	4.63	4.63	1370.95	0.0000	
	G×E	9.04	0.11	33.46	0.0000	

RSm, RS content in raw milled rice; RSc, RS content in cooked rice; RSr, RS content in retrograded rice; RSa is equal to RSm–RSc; RSb is equal to RSr–RSc.

### Identification of QTL associated with different types of RS

**Figure 3:**
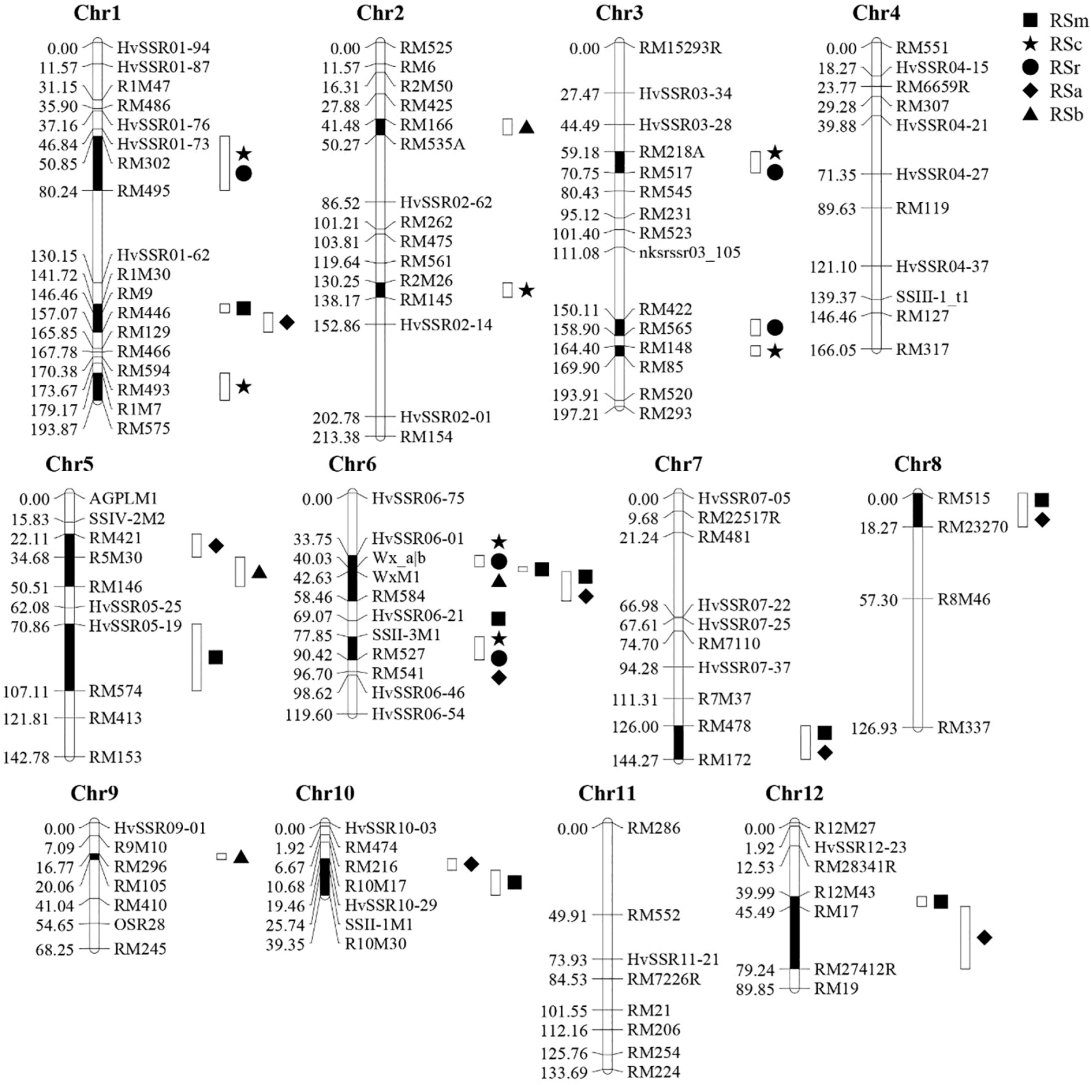
Genetic linkage map of different types of RS contents in RIL across two generations. RSm, RS content in raw milled rice; RSc, RS content in cooked rice; RSr, RS content in retrograded rice; RSa is equal to RSm–RSc; RSb is equal to RSr–RSc.

Next, 833 molecular markers were used to detect parental genetic polymorphism. And 123 pairs identified polymorphic markers were further employed to genotyping RILs. The genotyping results were consistent across both genetic generations. The constructed genetic map spanned a total length of 1635.23 cM, with an average marker spacing of 13.29 cM and a minimum inter-marker distance of 0.53 cM. A total of 33 QTLs associated with RSm, RSc, RSr, RSa, and RSb were identified through co-localization analysis of environmental traits across two generations (F_8_ and F_11_) (**Figure 3**). Nine QTLs related to RSm were detected, among which six proved to be significant (LOD > 3.0) and were mapped to chromosomes 1, 6, 7, and 8. Notably, three major-effect QTLs-*q2ERSm6.1*, *q2ERSm6.2*, and *q2ERSm6.3* were co-localized on chromosome 6. Each of these exhibited high LOD scores and explained 27.13%, 13.07% and 42.42% phenotypic variance, respectively (**Table 2**). And the genetic sources of *q2ERSm6.1* and *q2ERSm6.2* were from ‘Javanese Rice 22’, while *q2ERSm6.3* was from ‘CG133R’. In addition, seven QTLs related to RSc were detected. The *q2ERSc6.1* emerged as a major-effect QTL, positioned between markers HvSSR06–01 and Wx_a/b, accounting for 63.16% of the observed phenotypic variation. For RSr, a total of five QTLs were detected, some of which co-localized with QTLs for RSc. Among these, the PVE of *q2ERSr6.2* decreased to 14.09% from 17.82% of *q2ERSc6.2*, while the PVE changes of the other QTLs were relatively minor. Interestingly, *q2ERSm6.1*, *q2ERSc6.1* and *q2ERSr6.1* shared identical genomic intervals but exhibited opposite parental origins for their enhancer genes. Unlike *q2ERSm6.1*, the positive-effect alleles of both *q2ERSr6.1* and *q2ERSc6.1* were derived from ‘CG133R’. Furthermore, the QTLs for RSa shared similar marker intervals with those for RSm, with their positive-effect alleles also originating from the same parental source. Four QTLs related to RSb were detected, all exhibiting high contribution rates, with PVE ranging from 7.59% to 39.62%. Among them, the contribution rates of the environment interaction effects of *q2ERSb2.1* and *q2ERSb9.1* were significantly greater than their additive effects, indicating that the expression of these two QTLs is more susceptible to environmental influences. Moreover, for most QTLs—excluding *q2ERSr6.1* and those identified for RSb—both additive and environmental interaction effects contributed less than 1% of the phenotypic variation. This suggested their expression remained largely stable across the two generations, with minimal influence from environmental variation. Some QTLs on chromosome 6 are clustered in distribution. All five RS types detected QTLs linked to the Wx_a|b marker, which can explain 27.13 - 68.07% of the phenotypic variation. Except for RSb, the other four RS types detected QTLs associated with the SSIIaM1 marker, with phenotypic explanation rates ranging from 4.03% to 50.69%. Therefore, *Wx* and *SSIIa* were identified as the major QTLs regulating different RS subtypes in rice. Furthermore, this study identified several novel QTLs associated with RSm, RSc, and RSr, respectively (**Table 3**).

**Table 2:** The different types RS content related QTLs and their genetic effects detected from RILs across two generations.

QTLs locus	Marker interval	LOD	LOD(A)	LOD(AbyE)	PVE/%	PVE(A)/%	PVE(AbyE)/%	Add	AbyE1	AbyE2
*q2ERSm1.1*	R1M30-RM9	3.27	3.25	0.02	0.95	0.92	0.02	1.07	-0.18	0.18
*q2ERSm5.1*	HvSSR05-19-RM574	2.93	2.78	0.15	0.94	0.93	0.01	-0.99	-0.09	0.09
*q2ERSm6.1*	Wx_a|b-WxM1	39.40	35.74	3.66	27.34	27.13	0.21	8.33	-0.73	0.73
*q2ERSm6.2*	WxM1-RM584	30.75	30.52	0.23	13.15	13.07	0.08	6.51	-0.50	0.50
*q2ERSm6.3*	SSIIaM1-RM527	55.74	46.68	9.06	42.42	42.00	0.41	-6.76	-0.67	0.67
*q2ERSm7.1*	RM478-RM172	3.70	3.65	0.05	1.23	1.23	0.00	-1.15	0.00	0.00
*q2ERSm8.1*	RM515-RM23270	3.65	3.62	0.02	1.10	1.06	0.03	1.08	-0.18	0.18
*q2ERSm10.1*	SSIIcM1-R10M30	2.02	1.71	0.31	0.62	0.51	0.12	-0.76	0.37	-0.37
*q2ERSm12.1*	R12M43-RM17	2.02	1.98	0.04	0.63	0.63	0.00	0.81	0.02	-0.02
*q2ERSc1.1*	RM302-RM495	2.80	1.60	1.20	1.91	1.31	0.60	0.04	-0.03	0.03
*q2ERSc1.2*	R1M7-RM575	2.77	2.74	0.03	2.19	2.05	0.15	-0.05	-0.01	0.01
*q2ERSc2.1*	R2M26-RM145	2.73	2.68	0.05	2.06	2.06	0.01	-0.05	0.00	0.00
*q2ERSc3.1*	RM218A-RM517	2.63	2.61	0.01	2.02	1.99	0.03	0.05	0.01	-0.01
*q2ERSc3.2*	RM148-RM85	3.32	3.04	0.28	2.29	2.26	0.02	-0.06	0.01	-0.01
*q2ERSc6.1*	HvSSR06-01-Wx_a|b	42.38	36.83	5.55	64.16	64.08	0.08	0.39	0.01	-0.01
*q2ERSc6.2*	SSIIaM1-RM527	19.04	18.63	10.40	17.82	17.80	0.02	0.10	-0.01	0.01
*q2ERSr1.1*	RM302-RM495	2.66	0.92	1.74	1.20	0.94	0.26	0.05	-0.03	0.03
*q2ERSr3.1*	RM218A-RM517	2.30	1.85	0.45	1.89	1.80	0.09	0.06	0.01	-0.01
*q2ERSr3.2*	RM422-RM565	2.61	1.45	1.16	1.50	1.47	0.03	-0.06	0.01	-0.01
*q2ERSr6.1*	HvSSR06-01-Wx_a|b	43.15	30.01	13.14	68.07	66.94	1.14	0.53	0.07	-0.07
*q2ERSr6.2*	SSIIaM1-RM527	15.21	13.98	11.22	14.09	14.03	0.05	0.10	0.01	-0.01
*q2ERSa1.1*	RM9-RM446	4.06	4.02	0.03	1.55	1.55	0.00	1.21	-0.02	0.02
*q2ERSa5.1*	RM421-R5M30	2.40	2.39	0.01	0.99	0.96	0.02	-0.94	0.14	-0.14
*q2ERSa6.1*	WxM1-RM584	39.93	36.63	3.30	29.95	29.75	0.20	9.02	-0.73	0.73
*q2ERSa6.2*	SSIIaM1-RM527	53.45	45.26	8.19	51.16	50.69	0.46	-6.94	-0.66	0.66
*q2ERSa7.1*	RM478-RM172	4.55	4.51	0.03	2.08	2.07	0.00	-1.41	0.03	-0.03
*q2ERSa8.1*	RM515-RM23270	4.19	4.14	0.04	1.80	1.74	0.05	1.28	-0.22	0.22
*q2ERSa10.1*	HvSSR10-29-SSIIcM1	2.57	2.38	0.19	1.09	0.98	0.12	-1.01	0.35	-0.35
*q2ERSa12.1*	RM17-RM27412R	3.26	3.22	0.04	1.27	1.27	0.00	1.08	0.00	0.00
*q2ERSb2.1*	RM166-RM535A	2.25	0.90	1.35	10.73	2.98	7.75	-0.03	-0.04	0.04
*q2ERSb5.1*	R5M30-RM146	2.03	1.72	0.31	7.59	6.17	1.42	0.04	0.02	-0.02
*q2ERSb6.1*	HvSSR06-01-Wx_a|b	9.86	7.34	2.52	39.62	34.00	5.62	0.12	0.05	-0.05
*q2ERSb9.1*	RM296-RM105	2.89	0.65	2.24	11.58	2.34	9.25	0.02	0.05	-0.05

LOD, LOD score for all effects; LOD (A), LOD score for additive effects; LOD (AbyE), LOD score for additive by environment effects; PVE, phenotypic variation explained by all effects; PVE (A), phenotypic variation explained by additive effect; PVE (AbyE), phenotypic variation explained by additive by environment effect; Add, additive effect; AbyE1, additive effect of QTL for F_8_; AbyE2, additive effect of QTL for F_11_.

**Table 3:** The novel QTLs of different RS types.

QTLs locus	Maker interval	LOD value	PVE/%	Add
*q2ERSm1.1*	R1M30-RM9	3.27	0.95	1.07
*q2ERSm8.1*	RM515-RM23270	3.65	1.10	1.08
*q2ERSm12.1*	R12M43-RM17	2.02	0.63	0.81
*q2ERSc1.1*	RM302-RM495	2.80	1.91	0.04
*q2ERSc1.2*	R1M7-RM575	2.77	2.19	-0.05
*q2ERSc2.1*	R2M26-RM145	2.73	2.06	-0.05
*q2ERSc3.1*	RM218A-RM517	2.63	2.02	0.05
*q2ERSc3.2*	RM148-RM85	3.32	2.29	-0.06
*q2ERSr1.1*	RM302-RM495	2.66	1.20	0.05
*q2ERSr3.1*	RM218A-RM517	2.30	1.89	0.06
*q2ERSr3.2*	RM422-RM565	2.61	1.50	-0.06
*q2ERSa5.1*	RM421-R5M30	2.40	0.99	-0.94
*q2ERSa8.1*	RM515-RM23270	4.19	1.80	1.28
*q2ERSa12.1*	RM17-RM27412R	3.26	1.27	1.08
*q2ERSb5.1*	R5M30-RM146	2.03	7.59	0.04
*q2ERSb9.1*	RM296-RM105	2.89	11.58	0.02

LOD, LOD score for all effects; PVE, phenotypic variation explained by all effects; Add, additive effect.

### The genetic effects of two major QTLs on vairous RS types

**Figure 4:**
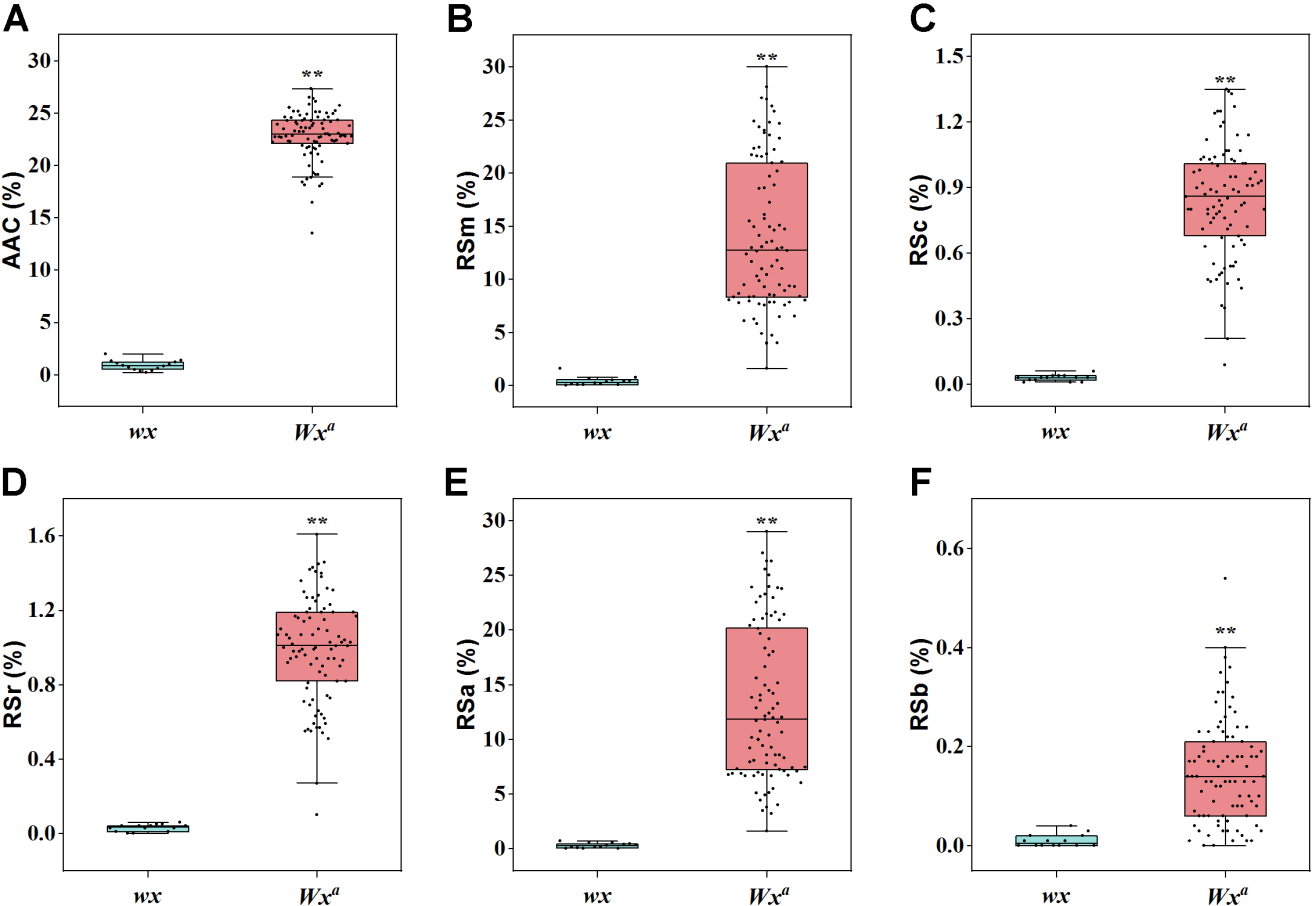
Genetic regulation of different RS types by *Wx* alleles. **(A)** AAC, apparent amylose content; **(B)** RSm, resistant starch content in milled rice; **(C)** RSc, resistant starch content in cooked rice; **(D)** RSr, resistant starch content in retrograded rice; **(E)** RSa is equal to RSm–RSc; **(F)** RSb is equal to RSr–RSc. One-way ANOVA, ^**^ indicates *P* < 0.01.

**Figure 5:**
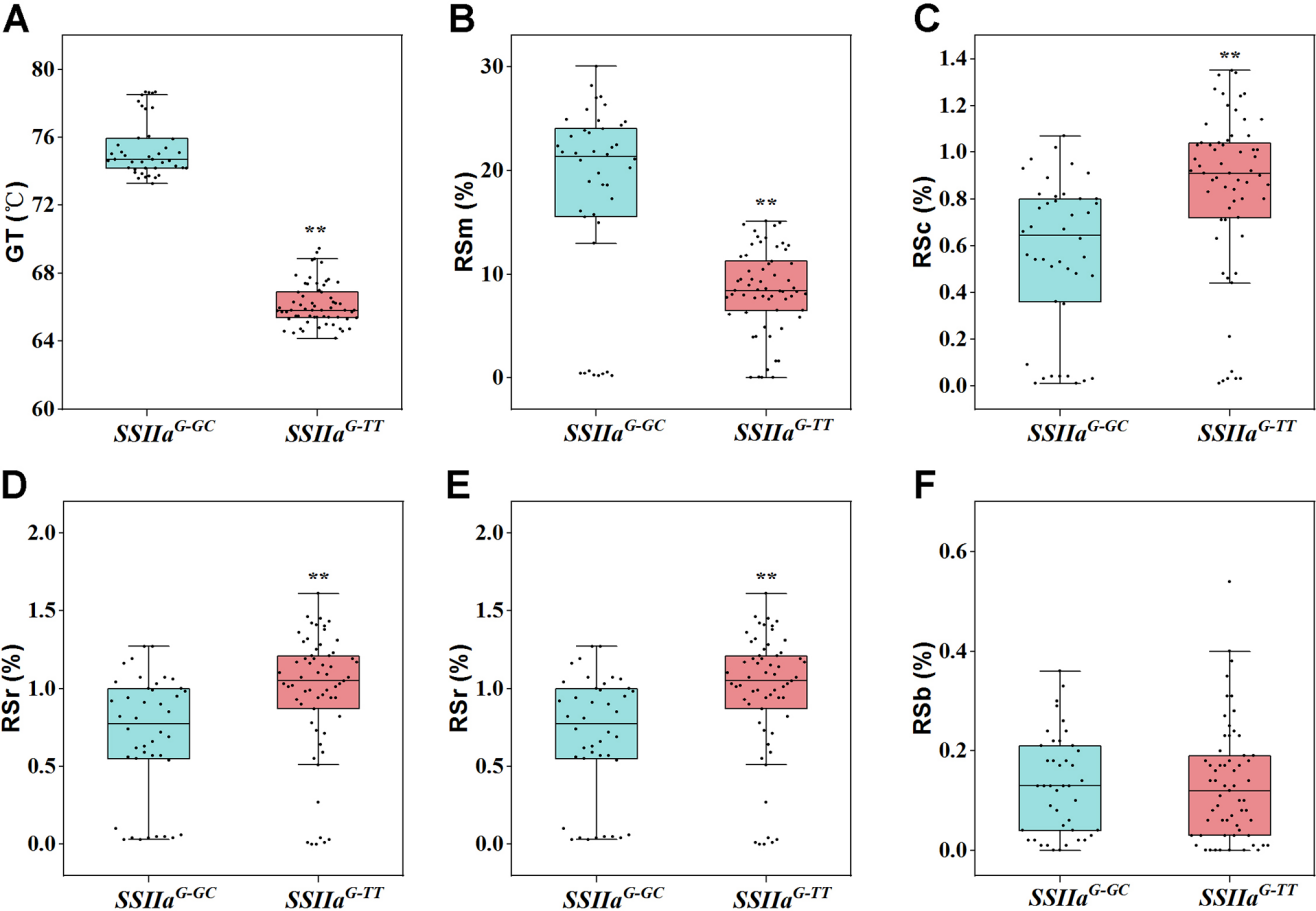
Genetic regulation of different RS types by *SSIIa* alleles. **(A)** GT, gelatinization temperature; **(B)** RSm, resistant starch content in milled rice; **(C)** RSc, resistant starch content in cooked rice; **(D)** RSr, resistant starch content in retrograded rice; **(E)** RSa is equal to RSm–RSc; **(F)** RSb is equal to RSr–RSc. One-way ANOVA, ^**^ indicates *P* < 0.01.

**Figure 6:**
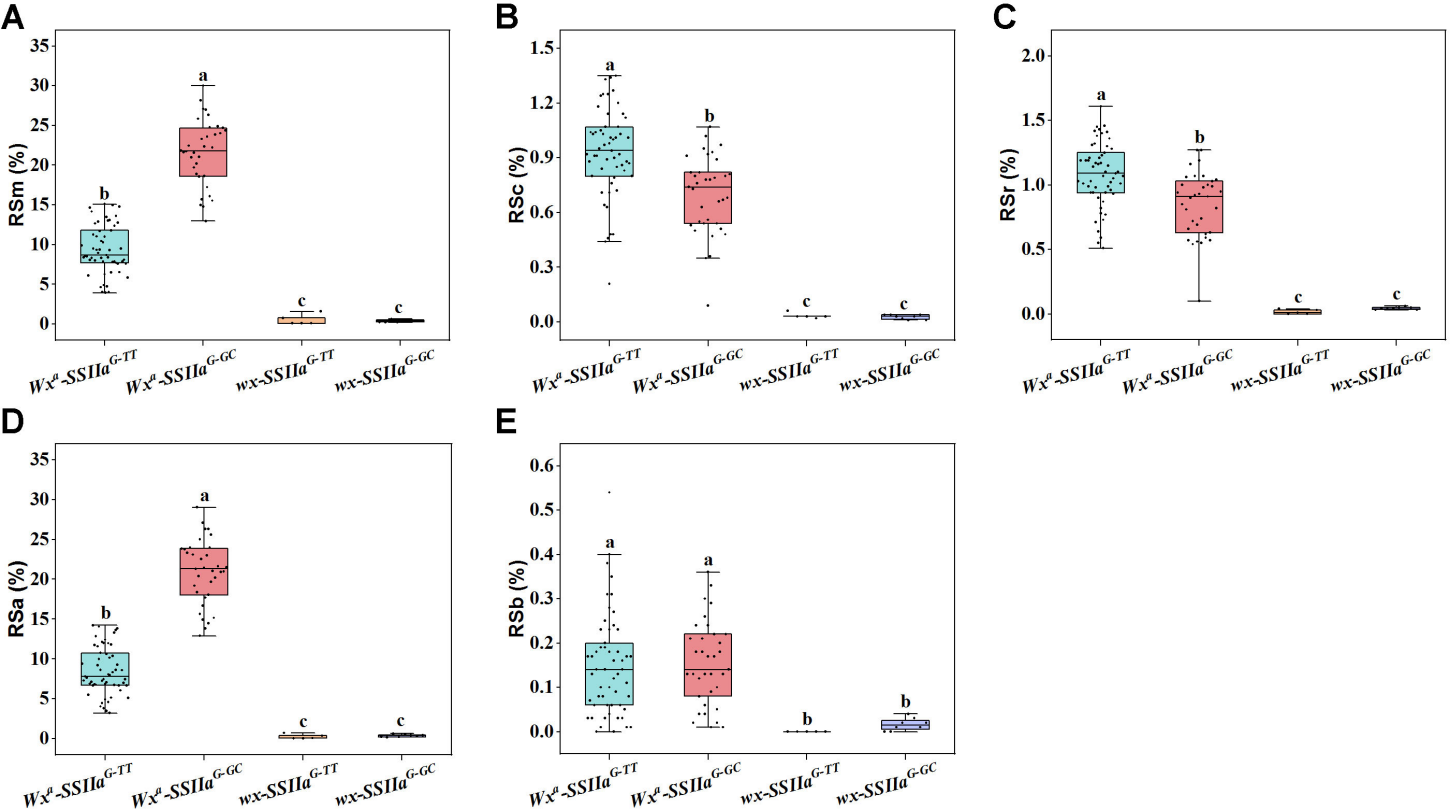
Genetic regulation of different RS types by the combined haplotypes of *Wx* and *SSIIa*. **(A)** RSm, resistant starch content in milled rice; **(B)** RSc, resistant starch content in cooked rice; **(C)** RSr, resistant starch content in retrograded rice; **(D)** RSa is equal to RSm–RSc; **(E)** RSb is equal to RSr–RSc. One-way ANOVA, Lowercases indicate significance at *P* < 0.05 (two-tail).

To further investigate the effects of the two major QTLs (*Wx* and *SSIIa*) on the various RS types, we detected the key functional variation sites of *Wx* and *SSIIa* in the two parents and the RIL F_11_ population (**Supplementary Table S2**). Two allelic variants of *Wx* were identified in the F_11_ population: *Wx^a^* (originated from CG133R) and *wx* (originated from Javanica 22), corresponding to the high AAC subpopulation and the glutinous rice subpopulation (AAC<2%), respectively (**Figure 4A**). Consistently, The RSm, RSc, RSr, RSa, and RSb in the *Wx^a^Wx^a^* genotype were greatly higher than those in the *wxwx* genotype, with average increases of 13.69%, 0.81%, 0.96%, 12.98%, and 0.14%, respectively (**Figures 4B–F**). According to the polymorphism of *SSIIa* gene, the F_11_ populations were divided into two types: *SSIIa^G-TT^* haplotype (derived from Javanica 22) and *SSIIa^G-GC^* haplotype (derived from CG133R). The *SSIIa^G-TT^* haplotype regulates low GT, while the *SSIIa^G-GC^* regulates high GT (**Figure 5A**). The average RSm of *SSIIa^G-GC^* (17.75%) was significantly higher than that of *SSIIa^G-TT^* (8.45%) (**Figure 5B**). In contrast, both RSc and RSr obviously decreased in *SSIIa^G-TT^* compared to *SSIIa^G-GC^* (*P* < 0.01), with average reductions of 0.28% and 0.27%, respectively (**Figures 5C, D**). *SSIIa* affected RSa in a manner consistent with its effect on RSm, but did not affect RSb (**Figures 5E, F**). The polymorphsims of *Wx* and *SSIIa* in F_11_ population resulted in four gene combinations, including *Wx^a^-SSIIa^G-TT^*, *Wx^a^-SSIIa^G-GC^*, *wx-SSIIa^G-TT^*, *wx-SSIIa^G-GC^*. Under the *wx* allele background, all RS types reached minimal levels, and no significant difference was detected between *SSIIa^G-GC^* and *SSIIa^G-TT^* (**Figure 6**). This demonstrates that *Wx* acts as the master regulator of RS biosynthesis in rice. Conversely, in the *Wx^a^* background, *SSIIa^G-GC^* exhibited signally elevated levels of RSm and RSa but markedly reduced levels of RSc and RSr compared to *SSIIa^G-TT^* (**Figures 6A–D**). The RSb of SSIIa_G-GC_ and SSIIa_G-TT_ did not differ significantly (**Figure 6E**). The *Wx^a^-SSIIa^G-TT^* haplotype was thus identified as the primary target for high-RS rice breeding, given its ability to enhance RS content in both cooked and retrograded rice, thereby better meeting daily human nutritional requirements.

### Distribution of ECQs across two generations and its correlation with RS content

**Figure 7:**
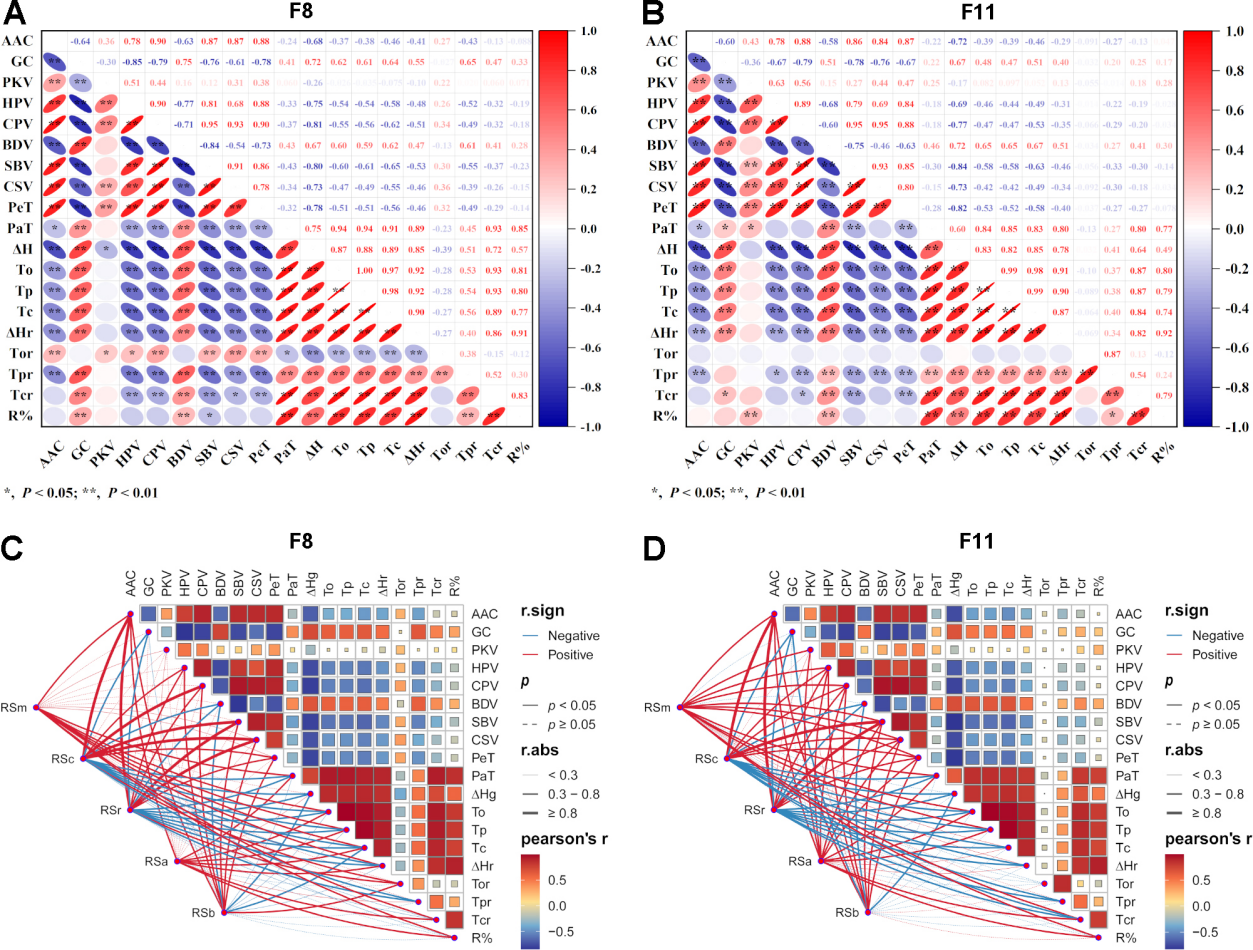
Correlation analysis of ECQs with RS. **(A)** Correlation analysis of ECQs in the RIL F_8_ population; **(B)** Correlation analysis of ECQs in the RIL F_11_ population; **(C)** Correlation analysis of ECQs with different RS types in the RIL F_8_ population; **(D)** Correlation analysis of ECQs with different RS types in the RIL F_11_ population. AAC, apparent amylose content; GC, gel consistency; To, onset temperature; Tp, peak temperature; Tc, conclusion temperature; ΔH, gelatinization enthalpy; Tor, retrograde onset temperature; TPr, retrograde peak temperature; Tcr, retrograde conclusion temperature; ΔHr, retrograde enthalpy change; R%, retrogradation rate; PKV, peak viscosity; HPV, hot paste viscosity; CPV, cool paste viscosity; BDV, breakdown value; SBV, setback value; CSV, consistence value; PeT, peak time; PaT, pasting temperature.

In RIL F_8_ and F_11_, bidirectional overdominance was observed for all ECQs traits except AAC and thermal properties (To, Tp, Tc). Most traits exhibited moderate to strong variation, with pronounced variation (CV > 20%) in AAC, GC, HPV, CPV, BDV, SBV, CSV, ΔH, ΔHr, and R% (**Supplementary Table S3**). The influence of environment on these traits was more significant than that of the RS. Marked differences between two generations were detected on AAC, PKV, HPV, PaT, and the thermal and retrogradation properties. Distributions for RVA parameters (except BDV) were similar across generations (**Supplementary Figure S3**), as were those for thermal and retrogradation properties (**Supplementary Figure S4**). A notable inverse pattern was found, however, whereby the F_8_ generation had lower thermal properties but higher retrogradation properties than F_11_, indicating a strong environmental effect on these specific traits. Correlation analysis among the ECQs yielded consistent results between the two generations (**Figures 7A, B**). AAC exhibited significant positive correlations with all RVA parameters (except BDV), whereas it correlated negatively with GC, thermal properties, and retrogradation properties. A significant positive correlation was observed among the thermal and retrogradation properties. Conversely, all RVA parameters, except BDV, showed significant negative correlations with both the thermal and retrogradation properties. Furthermore, most ECQs indicators displayed significant correlations with the RS content of various types. However, correlations between RS types and certain quality indicators varied across generations (**Figures 7C, D**), primarily due to environmental effects on ECQs. Whereas RSm was not significantly associated with viscosity parameters in the F_8_ population (**Figure 7C**), all five RS types in the F_11_ population showed greatly positive correlations with HPV, CPV, SBV, CSV, and PeT (**Figure 7D**). In addition, the AAC was obviously positively correlated with all five RS types across two generations. In contrast, GC and BDV showed negative correlations with RSc, RSr, and RSb. The thermal parameters (To, Tp, Tc, ΔH) and retrogradation properties (ΔHr, R%) were positively correlated with RSm and RSa, while exhibiting negative correlations with RSc and RSr. Based on this, there are superior haplotypes in the RIL population with high RSc and RSr and low GT and R%. Overall, RSc and RSr had significant correlations with more ECQs traits than RSm, RSa, and RSb, which provided a direction for improving the ECQs of rice with high RSc and RSr.

### Combined effects of *Wx* and *SSIIa* on ECQs

**Figure 8:**
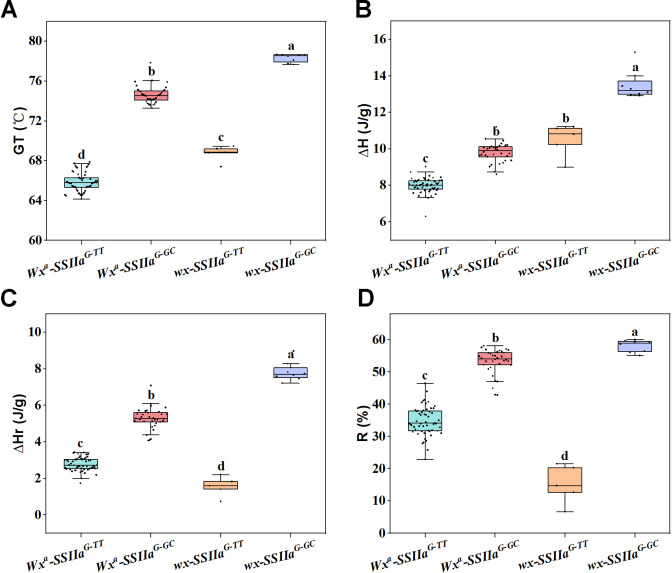
Genetic regulation of gelatinization and retrogradation properties by the combined haplotypes of *Wx* and *SSIIa*. **(A)** GT, gelatinization temperature; **(B)** ΔH, gelatinization enthalpy; **(C)** ΔHr, retrograde enthalpy change; **(D)** R%, retrogradation rate. One-way ANOVA, Lowercases indicate significance at *P* < 0.05 (two-tail).

**Figure 9:**
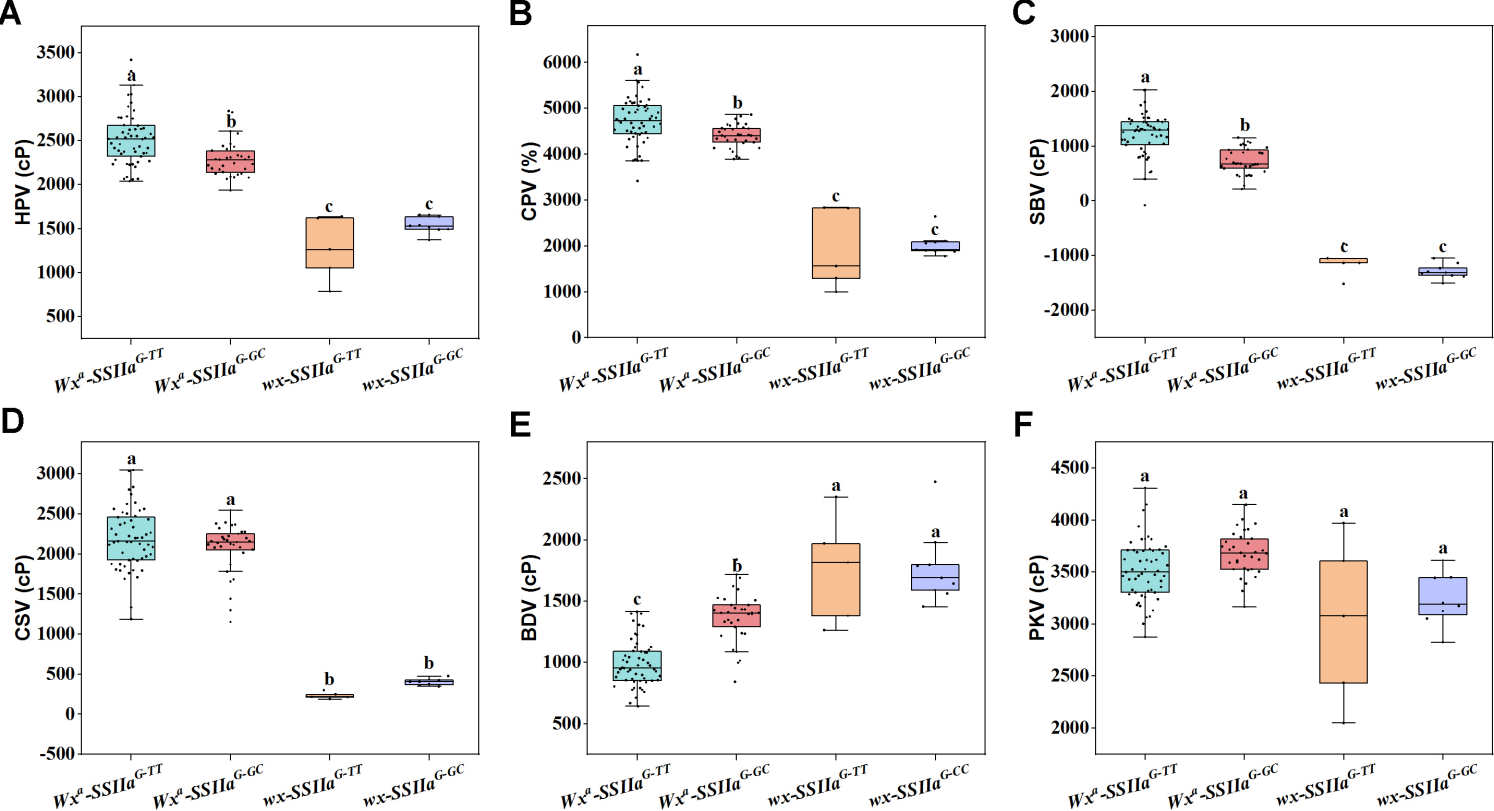
Genetic regulation of viscosity properties by the combined haplotypes of *Wx* and *SSIIa*. **(A)** HPV, hot paste viscosity; **(B)** CPV, cool paste viscosity; **(C)** SBV, setback value; **(D)** CSV, consistence value; **(E)** BDV, breakdown value; **(F)** PKV, peak viscosity. One-way ANOVA, Lowercases indicate significance at *P* < 0.05 (two-tail).

To identify the genetic factor of the significant correlations between different RS types and quality traits in the RIL F_11_ population, we further evaluated the effects of different haplotypes *Wx* and *SSIIa* on ECQs, particularly gelatinization, retrogradation, and viscosity properties. The results demonstrated significant differences in both the thermal and retrogradation properties among the four haplotypes of *Wx* and *SSIIa*. *Wx^a^-SSIIa^G-TT^* showed the lowest GT and ΔH compared to the other three haplotypes (**Figures 8A, B**). In addition, the ΔHr and R% of *Wx^a^-SSIIa^G-TT^* were significantly lower than those of *Wx^a^-SSIIa^G-GC^* by an average of 47.41% and 35.15%, respectively (**Figures 8C, D**). Moreover, significant variations in starch viscosity properties -- including HPV, CPV, SBV, CSV, and BDV -- were associated with different haplotypes (**Figures 9A–E**), with the exception of PKV, which showed no significant variation (**Figure 9F**). Comparing with other haplotypes, the *Wx^a^-SSIIa^G-TT^* haplotype exhibited the highest HPV, CPV, and SBV, along with the lowest BDV (*P* < 0.05). Therefore, *Wx^a^-SSIIa^G-TT^* haplotype improved the rice ECQs by lowering GT, preventing retrogradation and enhancing viscosity properties. In summary, the significant correlations between RS content and ECQs were driven by the pleiotropic regulation of *Wx* and *SSIIa*. Importantly, *Wx^a^-SSIIa^G-TT^* haplotype demonstrated the beneficial potential of this regulation by simultaneously increasing RSc and RSr content and improving the rice ECQs.

## Discussion

### Genetic and environmental regulation and formation mechanisms of different RS subtypes in rice

The RSm is primarily RS2, but cooking drastically reduces its content, leaving only RS5—formed by amylose-lipid complexes (Ding et al., 2019). Following 7 days of retrogradation, the slight increase of RS content is attributable to the formation of RS5. Therefore, RSa (RSm - RSc), RSc, and RSb (RSr - RSc) can be used to represent the three RS subtypes-RS2, RS5 and RS3, respectively (You et al., 2022). This study found that rice primarily contained RS2, with a lower content of RS5, and also produced limited RS3 during the retrogradation of gelatinized starch. RS content is a typical quantitative trait governed by a genetic architecture comprising a few major genes and numerous minor genes. The biosynthetic mechanisms of different RS subtypes in rice are extremely complex and their regulatory networks are still elusive (Liang et al., 2024). This study found that all RS types exhibited varying degrees of bidirectional transgressive segregation in the RILs, with RSm manifesting the most extreme phenotype. This phenomenon is likely attributable to epistatic interactions among the alleles derived from the two parental genotypes within the RIL population. All five RS types were influenced by the genotype-environment interaction. Additionally, except for RSc, the other four RS types were also affected by environmental effects. However, the broad-sense heritability of RSm, RSc, RSr, and RSa was greater than 0.7, indicating a predominant genetic control and agreeing with previous reports (Biselli et al., 2019). In contrast, RSb (RS3) showed highly significant inter-generational differences with a broad-sense heritability below 0.5. This indicates that it is influenced more by environmental factors other than genotype. RS3 content was associated with both internal and external factors of starch retrogradation. Internal factors include the amylose-to-amylopectin ratio, lipid content, and amylopectin branching degree, while external factors encompass temperature, storage duration, and cooling rate (Li et al., 2016; Vamadevan and Bertoft, 2018; Bao et al., 2020). Therefore, the formation of RS3 is highly complex, and its mechanisms have not yet been fully elucidated.

### Discovery of novel QTLs for RS and analysis of synergistic effects between *Wx* and *SSIIa* genotypes

Currently, more research has focused on identifying genes related to RS in raw rice flour. In contrast, QTL mapping for RS content in cooked and retrograded rice is limited. A GWAS found that a total of 11 significant associations with RS were observed on chromosomes 1, 2, 3 5, 6, 8, and 11 (Biselli et al., 2019). Segregation analysis of a population derived from the high-RS mutant *RS111* revealed that SSR markers on chromosomes 8 and 6 were associated with RS content (Collaborating, 2008). In this study, multiple QTLs for all types of RS were detected on chromosomes 1, 3 and 6. Besides, additional QTLs for RSm and RSa were detected on chromosomes 7 and 8. Notably, two QTLs for RS content, designated qRS7–1 and qRS7-2, were previously mapped to chromosome 7 in an F_2–3_ population from a cross between rice genotypes Gongmi No. 3 and Diantun 502 (Zeng et al., 2016). Consistent with this, the QTLs identified in our study—qRSm7.1, qRSa7.1, q2ERSm7.1, and q2ERSa7.1—all overlapped with the reported qRS7–2 locus. Moreover, two major-effect QTLs (*Wx* and *SSIIa*) capable of influencing five types of RS content were both detected on chromosome 6. Studies have revealed that the key genes mapped for RS regulation were predominantly those involved in starch synthesis. Key SSRGs-including *Wx*, *SSIIa*, *SSIIIa*, and *BEIIb* are known to influence RS formation. Furthermore, combinatorial effects of these SSRGs on RS content have been documented in rice (Shen et al., 2022). The additive effect of *SSIIa* together with *Phosphofructokinase* (*pfkB*) explained RS phenotype variation of 19% by TGAS (Parween et al., 2020). The interaction between *Wx* and *SSIIa* significantly affected rice RS content (Zhou et al., 2016; You et al., 2022). Moreover, *Wx* had significant interactions with *SSIIa*, *SSI*, *SSIIb* and *SSIVb* on RSm, but only the dominant interactions with *SSIIb* and *SSI* on RSc and RSr (Liang et al., 2024). Our findings further demonstrate that *Wx* and *SSIIa* have differential synergistic effects on different RS types: *Wx^a^-SSIIa^G-GC^* genotype enhanced RSm, while *Wx^a^-SSIIa^G-TT^* boosted both RSc and RSr. In addition to the major-effect QTLs, several minor QTLs identified in this study also co-located with previously reported loci. Specifically, *q2ERSb2.1* corresponded to the *qRS-2* interval. Both *qRSm5.1* and *qRSa5.1* fell within the *qRS-5-1* region, while *q2ERSm5.1* aligned with *qRS-5-2*. Furthermore, *q2ERSm10.1* and *q2ERSa10.1* were positioned near the *qRS-10* locus (Wang, 2015). Furthermore, this study uncovered several novel QTLs. *q2ERSb5.1* and *q2ERSb9.1*, with contribution rates of 7.59–11.58%, were stably detected across both environments, making them suitable for subsequent fine mapping and marker-assisted breeding (**Table 3**).

### Identification of key genetic background (*Wx^a^-SSIIa^G-TT^*) for synergistic improvement of RS content and ECQs in rice

RS in rice is closely associated with amylose content, thus high-RS germplasm requires high amylose levels (Zhou et al., 2016; Shen et al., 2022). In this study, all five types of RS showed a significantly positive correlation with AAC across two growing seasons. The correlation between RSm and AAC was weaker than that between RSc and AAC, aligning with the results reported by Ding et al. (2019). Similarly, a study observed a significant positive correlation between AAC and both RS2 and RS3 (Luo et al., 2023), which was in agreement with the results of the present study. *Wx^a^* regulates the synthesis of high AAC in rice endosperm, thereby enhancing the production of RSm. Additionally, the fine structure and chain length distribution of amylopectin regulated by *SSIIa* also affect the RS content. The functional *SSIIa* produces high-crystallinity starch that is more resistant to enzymatic digestion, leading to a higher RS content. (Bao et al., 2017) Downregulation of *SSIIa* in rice significantly increases the short A and B1 chains of amylopectin (6 ≤ DP ≤ 12) and reduces the medium and long-type B1 chains (13 ≤ DP ≤ 24) (Butardo et al., 2011; Zhang et al., 2020). Thus, *SSIIa^G-GC^* promotes the synthesis of long-type B1 chains and high crystallinity by expressing highly active SSIIa, resulting in elevated RSm content. However, the formation of RSc may be related to the content of amylopectin short chains during starch cooking. The *SSIIa^G-TT^* genotype, which possesses a greater number of amylopectin short chains, consequently increase RSc. As for rice quality, high AAC often leads to inferior ECQs. Therefore, it is particularly challenging to improve the palatability of high-RS rice to meet the taste preferences of individuals managing blood sugar levels. This study further revealed that the gelatinization and retrogradation properties of rice showed a significant positive correlation with RSm, but a significant negative correlation with both RSc and RSr. This offers a potential strategy for improving the ECQs of high-RS rice by targeting reduced GT and retrogradation. Although previous studies have identified that the mutant *RS111*, which has a high RSc value, exhibited significantly lower GT and ΔH compared to the wild type (Yang et al., 2005), other research has reported a significant positive correlation between RS3 and both ΔH and R% (Miura et al., 2021). These findings collectively indicated that the relationship between RSc and the gelatinization/retrogradation properties in rice was influenced by the genetic background. Our study further established that the *Wx^a^-SSIIa^G-TT^* haplotype was the key genetic background conferring both high RSc and low GT/R% in rice. The *Wx^a^-SSIIa^G-TT^* creates a genetic background that features high AAC alongside reduced starch crystallinity and an increased proportion of short branches. Thus, it enables the concurrent achievement of high enzymatic resistance and superior pasting properties. This finding provides a viable breeding strategy for simultaneously increasing RS content in cooked rice and improving its ECQs.

### Correlation analysis of rice RVA profile characteristics with RS subtypes and breeding applications of the key haplotype (*Wx^a^-SSIIa^G-TT^*)

RVA profile characteristics are important indicators that characterize the viscosity changes of rice during cooking. Previous studies have revealed uncertainties in the correlation between RS and viscosity properties. One study found that RS was significantly positively correlated with PKV and BDV (Nakamura et al., 2016). However, other study reported a significant negative correlation between RS and PKV and BDV (Chen et al., 2017). RS2 was positively correlated with HPV and CPV. In contrast, RS3 exhibited strong negative correlations with viscosity properties (Ding et al., 2019). We proposed that the discrepancies in these correlation results arose from genetic differences in the research materials used and variations in RS subtypes. In the present study, only RSm and RSa showed a significant positive correlation with PKV, whereas RSc and RSr exhibited no significant correlation with PKV. Additionally, RSc, RSr, and RSb were signally negatively correlated with BDV and markedly positively correlated with HPV, CPV, SBV and SBV. Haplotype analysis revealed that the *Wx^a^-SSIIa^G-TT^* haplotype was a genetic factor leading to high HPV, CPV, SBV, and low BDV. Since *Wx^a^-SSIIa^G-TT^* also regulates high RSc and RSr content, it consequently results in an obvious positive correlation with SBV and a significant negative correlation with BDV. Furthermore, the RVA pasting properties of rice are significantly influenced by environmental conditions. This environmental variability caused the differing correlation results observed between viscosity traits and RSm in the F_8_ and F_11_ generations. In summary, improving the viscosity properties of high-RS rice relies not only on the selection of key gene haplotypes but also on the adjustment of cultivation environments. Specifically, the *Wx^a^-SSIIa^G-TT^*, compared to *Wx^a^-SSIIa^G-GC^*, could enhance both rice viscosity and RSc content, making it a superior haplotype for application in breeding programs.

Overall, a total of 33 QTLs associated with RSm, RSc, RSr, RSa, and RSb were detected through co-localization analysis across two generations (F_8_ and F_11_) of RILs in present study. The *Wx* and *SSIIa* genes were identified as two major QTLs. Additionally, several novel minor QTLs were discovered, such as *q2ERSc3.2*, *q2ERSb5.1*, and *q2ERSb9.1*. In addition, the contents of the five RS types were closely correlated with ECQs. Specifically, while the thermal and retrogradation properties showed positive correlations with RSm and RSa, they were negatively correlated with RSc and RSr. Furthermore, RSc, RSr, and RSb were significantly negatively correlated with BDV but positively correlated with HPV, CPV, and SBV. Haplotype analysis revealed that *Wx^a^-SSIIa^G-GC^* regulated high RSm and RSa, while *Wx^a^-SSIIa^G-TT^* controlled high RSc and RSr. Meanwhile, the *Wx^a^-SSIIa^G-TT^* haplotype also contributed to starch properties characterized by low GT, low retrogradation, and high viscosity. Therefore, given its dual benefits of increasing cooked rice RS content and improving ECQs, *Wx^a^-SSIIa^G-TT^* haplotype was appropriate for breeding rice varieties with high RSc. These findings have identified potential minor-effect QTLs regulating RS, and further elucidated the relationship between rice ECQs and different RS types. This work provided new avenues for the targeted breeding of high-RSc rice and quality improvement.

## Data Availability

The original contributions presented in the study are included in the article/**Supplementary Material**. Further inquiries can be directed to the corresponding authors.
