# Supplementary material for: QTL mapping for different resistant starch subtypes identified a superior haplotype balancing high RS content and relatively good eating and cooking qualities in rice
Source: Front Plant Sci. 2026 Jan 27;17:1763165. doi: 10.3389/fpls.2026.1763165 (PMC12886350; doi:10.3389/fpls.2026.1763165)
Supplement: Supplementary file 1 [file DataSheet1.docx]

**Supplementary data**

**Table S1** Statistical analysis of different RS type of RILs across two generations.

**Table S2** Genotyping of *Wx* and *SSIIa* genes in parents.

**Table S3** Variation in eating and cooking qualities of RILs across two generations.

**Fig. S1.** The physical locations of molecular markers.

**Fig. S2.** The content of different types of RS in the parental lines.

**Fig. S3.** Distribution of viscosity properties in RILs across two generations.

**Fig. S4.** Distribution of gelatinization and retrogradation properties in RILs across two generations.

| **Table S1** Statistical analysis of different RS type of RILs across two generations |
| --- |
| \| Traits \| Year \| Min \| Max \| Mean ± SD \| CV% \| Skewness \| Kurtosis \| \| --- \| --- \| --- \| --- \| --- \| --- \| --- \| --- \| \| RSm (%) \| 2018 \| 0.05 \| 29.00 \| 10.95±8.58^A^ \| 78.36 \| 0.76 \| -1.00 \| \| 2021 \| 0.04 \| 30.03 \| 12.46±8.32^A^ \| 66.77 \| 0.35 \| -0.92 \| \| RSc (%) \| 2018 \| 0.00 \| 1.71 \| 0.75±0.39^BC^ \| 52.00 \| -0.56 \| -0.07 \| \| 2021 \| 0.01 \| 1.35 \| 0.75±0.36^C^ \| 48.00 \| -0.78 \| -0.17 \| \| RSr (%) \| 2018 \| 0.00 \| 2.21 \| 1.09±0.57^B^ \| 52.29 \| -0.35 \| -0.41 \| \| 2021 \| 0.01 \| 1.46 \| 0.89±0.42^BC^ \| 47.19 \| -0.96 \| -0.04 \| \| Rsa (%) \| 2018 \| 0.05 \| 28.00 \| 10.20±8.54^A^ \| 83.73 \| 0.78 \| -1.02 \| \| 2021 \| 0.03 \| 29.01 \| 11.70±8.22^A^ \| 70.26 \| 0.41 \| -0.97 \| \| RSb (%) \| 2018 \| 0.00 \| 1.28 \| 0.34±0.27^D^ \| 79.41 \| 0.96 \| 0.68 \| \| 2021 \| 0.00 \| 0.40 \| 0.14±0.10^E^ \| 71.43 \| 0.45 \| -0.50 \| |
| RSm, RS content in raw milled rice; RSc, RS content in cooked rice; RSr, RS content in retrograded rice; RSa is equal to RSm–RSc; RSb is equal to RSr–RSc. One-way ANOVA, uppercase letters indicate significant difference at *P* < 0.01. |

| **Table S2** Genotyping of *Wx* and *SSIIa* genes in parents |
| --- |
| \| Gene \| Maker name \| Primer \| Loci \| Genotype \| \| \| --- \| --- \| --- \| --- \| --- \| --- \| \| CG133R \| Javanica22 \| \| *Wx* \| Wx_a\|b \| F: CTCTGCTTGTGTTGTTCTGTTGT \| Intron1-1  T/G \| G/G \| T/T \| \| R: TTCCAGCCCAACACCTTACAG \| \| Wx M1 \| F: TGCAGAGATCTTCCACAGCA \| Exon2  23bp replicate \| -/- \| 23bp replicate \| \| R: GCTGGTCGTCACGCTGAG \| \| *SSIIa* \| SSIIa M1 \| F: CCAATACCGTAAACTAGCGACTATG \| Promoter  9bp indel \| -/- \| 9bp indel \| \| R: TACAGGTAGAATGGCAGTGGTG \| \| SSIIa M2 \| F: GGTTCTCGGTGAAGATGGC \| Exon8-864,865 TT/GC \| GC/GC \| TT/TT \| \| R: GTGGTCCCAGCTGAGGTCC \| \| SSIIa M3 \| F: CTCAACCAGCTCTACGCCAT \| Exon8-733 G/A \| G/G \| G/G \| \| R: GCTCTCCTTGTACTTGCGGT \| |

| **Table S3** Variation in eating and cooking qualities of RILs across two generations. |
| --- |
| \| Traits \| Year \| Parent \| \|  \| RIL \| \| \| \| \| \| --- \| --- \| --- \| --- \| --- \| --- \| --- \| --- \| --- \| --- \| \| CG133R \| J22 \|  \| Range \| Mean±SD \| CV% \| Skewness \| Kurtosis \| \| AAC (%) \| 2018 \| 23.51 \| 0.00 \|  \| 0.34~25.68 \| 19.07±7.16* \| 37.55 \| -1.82 \| 1.93 \| \| 2021 \| 17.32 \| 0.00 \|  \| 0~23.33 \| 16.54±6.45 \| 39.00 \| -1.90 \| 2.36 \| \| GC (mm) \| 2018 \| 7.90 \| 11.30 \|  \| 1.80~11.67 \| 6.35±3.01 \| 47.40 \| 0.03 \| -1.22 \| \| 2021 \| 2.82 \| 10.25 \|  \| 1.87~11.38 \| 5.83±2.72 \| 46.66 \| 0.46 \| -0.85 \| \| PKV (cP) \| 2018 \| 3321.00 \| 3551.00 \|  \| 2773.00~4739.00 \| 3676.55±352.17** \| 9.58 \| 0.47 \| 1.39 \| \| 2021 \| 3039.00 \| 3080.33 \|  \| 2432.00~4146.00 \| 3531.42±295.28 \| 8.36 \| -0.73 \| 1.49 \| \| HPV (cP) \| 2018 \| 2474.00 \| 1736.00 \|  \| 1053.00~3824.00 \| 2472.97±522.27* \| 21.12 \| -0.43 \| 0.36 \| \| 2021 \| 2266.00 \| 1544.33 \|  \| 1052.00~3289.00 \| 2288.82±416.53 \| 18.20 \| -0.63 \| 0.80 \| \| CPV (cP) \| 2018 \| 4674.00 \| 2224.00 \|  \| 1339.00~5667.50 \| 4295.53±1017.34 \| 23.68 \| -1.32 \| 1.09 \| \| 2021 \| 4284.00 \| 1997.67 \|  \| 1297.00~5605.00 \| 4219.88±992.45 \| 23.52 \| -1.56 \| 1.66 \| \| BDV (cP) \| 2018 \| 847.00 \| 1815.00 \|  \| 492.00~2786.00 \| 1203.58±494.55 \| 41.09 \| 0.98 \| 1.05 \| \| 2021 \| 773.00 \| 1536.00 \|  \| 642.00~2474.00 \| 1242.60±381.21 \| 30.68 \| 0.83 \| 0.74 \| \| SBV (cP) \| 2018 \| 1353.00 \| -1327.00 \|  \| -2022.50~2032.00 \| 618.97±960.00 \| 155.10 \| -1.21 \| 0.69 \| \| 2021 \| 1245.00 \| -1082.67 \|  \| -1692.00~1803.00 \| 688.46±899.17 \| 130.61 \| -1.46 \| 1.10 \| \| CSV (cP) \| 2018 \| 2200.00 \| 488.00 \|  \| 286.00~3032.00 \| 1822.55±613.30 \| 33.65 \| -0.89 \| 0.44 \| \| 2021 \| 2018.00 \| 453.33 \|  \| 245.00~2835.00 \| 1931.06±654.57 \| 33.90 \| -1.46 \| 1.29 \| \| PeT (min) \| 2018 \| 6.23 \| 4.40 \|  \| 3.53~6.63 \| 5.87±0.69 \| 11.75 \| -1.84 \| 2.97 \| \| 2021 \| 6.27 \| 4.45 \|  \| 3.47~6.60 \| 5.87±0.70 \| 11.93 \| -2.08 \| 3.56 \| \| PaT (℃) \| 2018 \| 72.48 \| 83.15 \|  \| 71.70~83.95 \| 76.68±3.85** \| 5.02 \| 0.23 \| -1.61 \| \| 2021 \| 72.60 \| 82.97 \|  \| 70.05~81.50 \| 74.88±3.51 \| 4.69 \| 0.26 \| -1.48 \| \| ∆H (J/g) \| 2018 \| 7.54 \| 14.60 \|  \| 5.38~15.10 \| 8.87±1.99 \| 22.44 \| 1.02 \| 0.72 \| \| 2021 \| 7.31 \| 15.96 \|  \| 6.30~15.28 \| 9.21±1.73 \| 18.78 \| 1.39 \| 1.87 \| \| To (℃) \| 2018 \| 62.08 \| 78.06 \|  \| 61.16~78.69 \| 67.79±5.83** \| 8.60 \| 0.39 \| -1.62 \| \| 2021 \| 60.64 \| 77.00 \|  \| 58.42~75.35 \| 65.12±5.58 \| 8.57 \| 0.41 \| -1.51 \| \| Tp (℃) \| 2018 \| 67.09 \| 81.44 \|  \| 65.95~81.62 \| 72.06±5.10** \| 7.08 \| 0.38 \| -1.61 \| \| 2021 \| 65.60 \| 80.97 \|  \| 64.15~78.66 \| 69.98±4.87 \| 6.96 \| 0.41 \| -1.50 \| \| Tc (℃) \| 2018 \| 72.69 \| 85.87 \|  \| 71.70~85.90 \| 77.38±4.49** \| 5.80 \| 0.42 \| -1.43 \| \| 2021 \| 70.99 \| 86.49 \|  \| 70.29~84.23 \| 75.52±4.24 \| 5.61 \| 0.46 \| -1.24 \| \| ∆Hr (J/g) \| 2018 \| 1.99 \| 6.81 \|  \| 1.30~7.10 \| 3.22±1.52** \| 47.20 \| 1.02 \| 0.15 \| \| 2021 \| 2.34 \| 8.61 \|  \| 0.75~8.96 \| 4.01±1.81 \| 45.14 \| 0.82 \| -0.18 \| \| Tor (℃) \| 2018 \| 44.12 \| 44.38 \|  \| 38.08~44.71 \| 41.53±1.75** \| 4.21 \| 0.12 \| -0.74 \| \| 2021 \| 43.02 \| 41.55 \|  \| 38.14~47.85 \| 42.91±1.99 \| 4.64 \| 0.39 \| 0.64 \| \| Tpr (℃) \| 2018 \| 51.91 \| 54.06 \|  \| 47.92~54.94 \| 51.24±1.48** \| 2.89 \| 0.10 \| -0.37 \| \| 2021 \| 51.40 \| 52.56 \|  \| 47.76~55.92 \| 51.98±1.56 \| 3.00 \| 0.12 \| 0.76 \| \| Tcr (℃) \| 2018 \| 59.62 \| 64.03 \|  \| 58.70~64.29 \| 61.30±2.01** \| 3.28 \| 0.33 \| -1.75 \| \| 2021 \| 59.19 \| 64.26 \|  \| 57.54~63.09 \| 60.42±1.70 \| 2.81 \| 0.10 \| -1.40 \| \| R (%) \| 2018 \| 26.23 \| 47.37 \|  \| 12.01~54.89 \| 35.23±9.01** \| 25.71 \| 0.03 \| -0.64 \| \| 2021 \| 32.35 \| 54.68 \|  \| 7.21~64.74 \| 42.56±12.44 \| 28.57 \| -0.11 \| -0.71 \| |
| AAC, apparent amylose content; GC, gel consistency; PKV, peak viscosity; HPV, hot paste viscosity; CPV, cool paste viscosity; BDV, breakdown value; SBV, setback value; CSV, consistence value; PeT, peak time; PaT, pasting temperature; ∆H, gelatinization enthalpy; To, onset temperature; Tp, peak temperature; Tc, conclusion temperature; ∆Hr, Enthalpy of retrogradation; Tor, onset temperature of retrogradation; Tpr, peak temperature of retrogradation; Tcr, conclusion temperature of retrogradation; R%, Retrogradation rate. One-way ANOVA, ^*^ and ^**^ indicate traits in two generations are significantly different at *P* < 0.05 and *P* < 0.01, respectively. |

|  |
| --- |
| **Fig. S1.** The physical locations of molecular markers. |

| 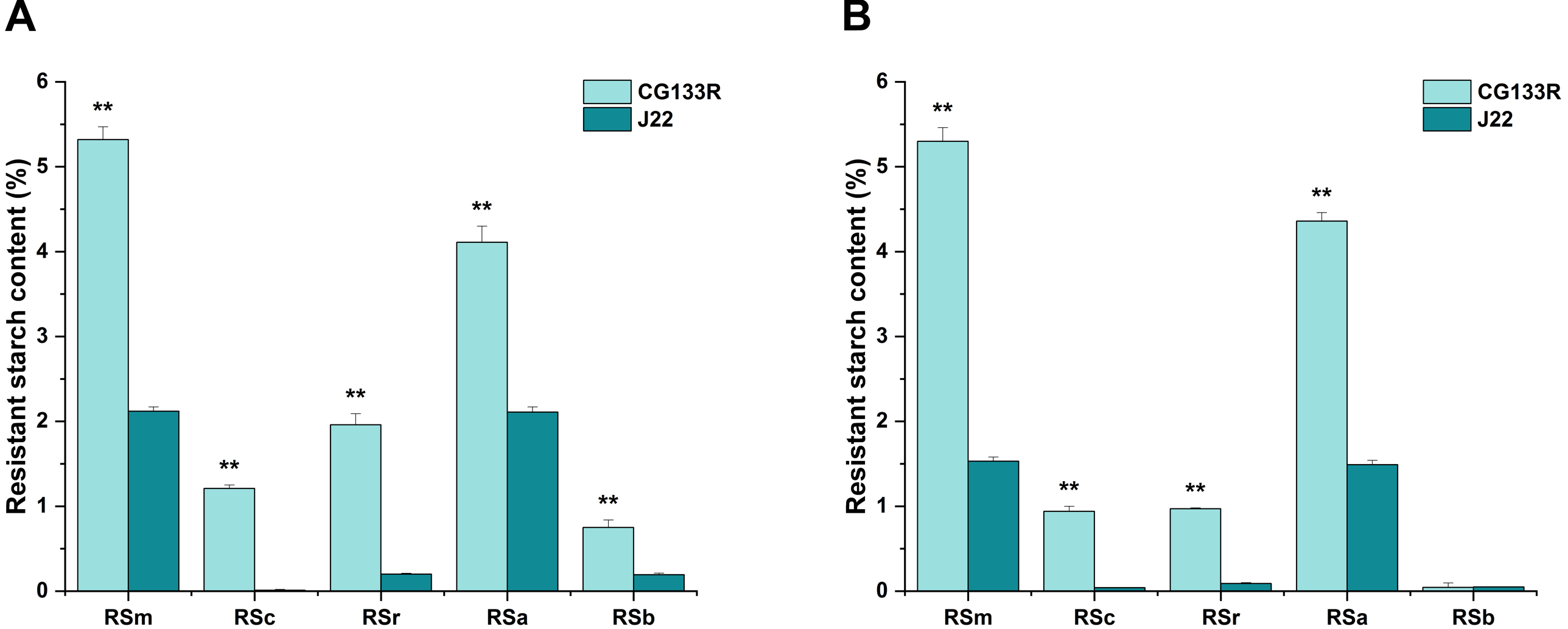 |
| --- |
| **Fig. S2.** The content of different types of RS in the parental lines. RSm, RS content in raw milled rice; RSc, RS content in cooked rice; RSr, RS content in retrograded rice; RSa is equal to RSm–RSc; RSb is equal to RSr–RSc. One-way ANOVA, ^*^ and ^**^ indicate traits in two generations are significantly different at *P* < 0.05 and *P* < 0.01, respectively. |

| 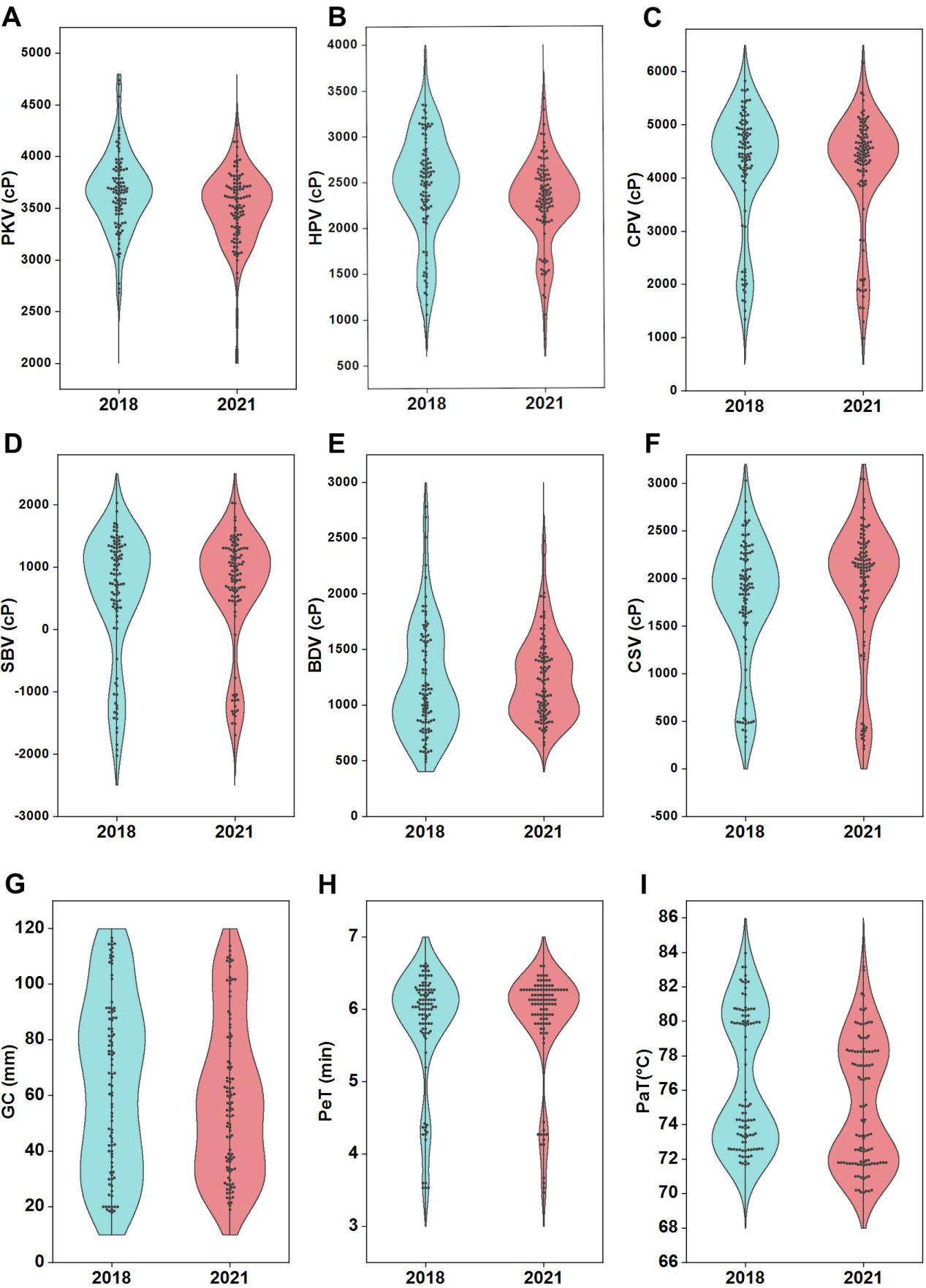 |
| --- |
| **Fig. S3.** Distribution of viscosity properties in RILs across two generations. (A) PKV, peak viscosity. (B) HPV, hot paste viscosity; (C) CPV, cool paste viscosity; (D) SBV, setback value; (E) BDV, breakdown value; (F) CSV, consistence value; (G) GC, gel consistency; (H) PeT, peak time; (I) PaT, pasting temperature. |

| 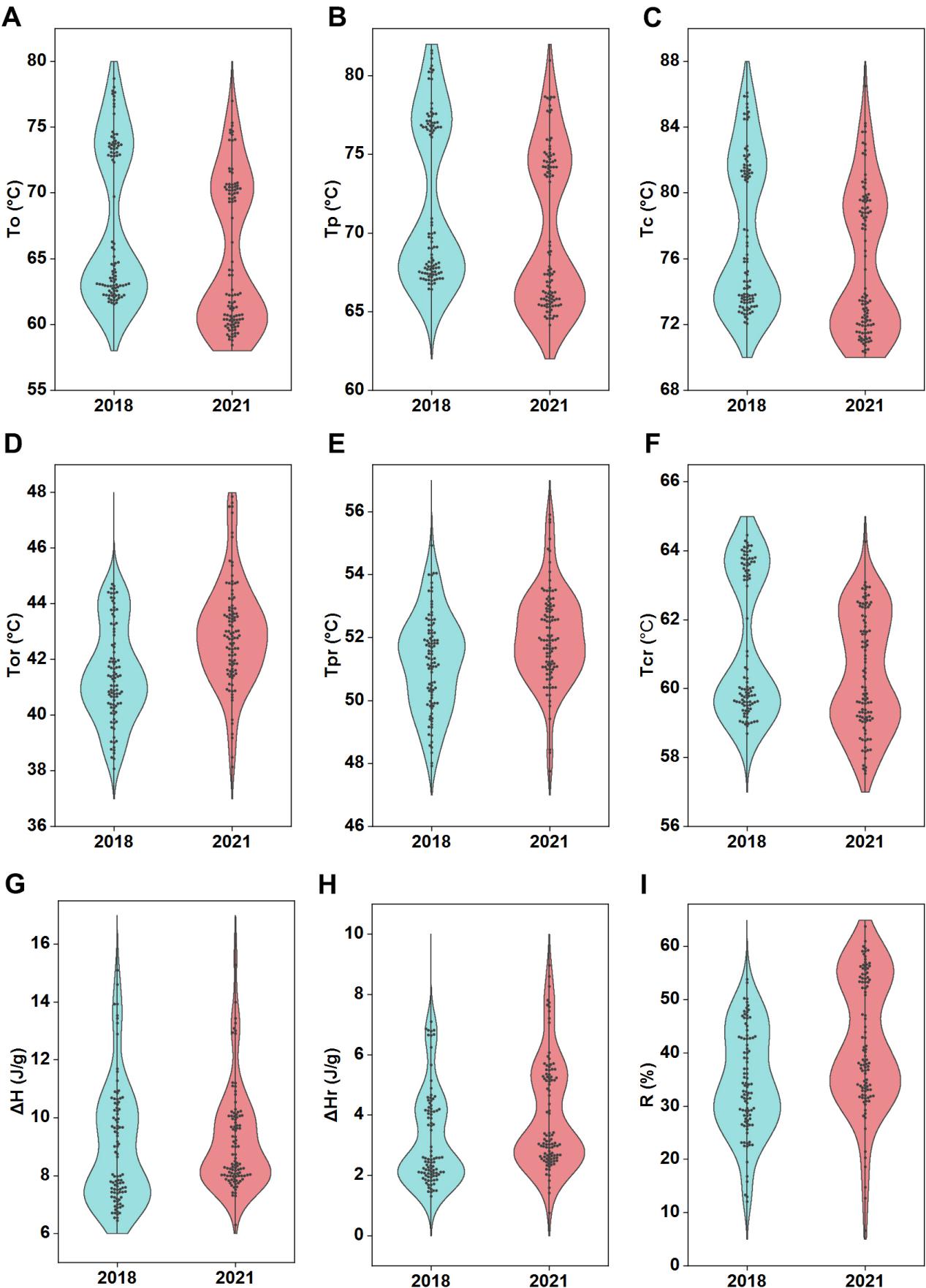 |
| --- |
| **Fig. S4.** Distribution of gelatinization and retrogradation properties in RILs across two generations. (A) To, onset temperature; (B) Tp, peak temperature; (C) Tc, conclusion temperature; (D) Tor, retrograde onset temperature; (E) Tpr, retrograde peak temperature; (F) Tcr, retrograde conclusion temperature; (G) ∆H, gelatinization enthalpy; (H) ∆Hr, retrograde enthalpy change; (I) R%, retrogradation rate. |
